# Emission control of entangled electrons in photoionisation of a hydrogen molecule

**DOI:** 10.1038/s41598-024-67465-0

**Published:** 2024-08-23

**Authors:** Farshad Shobeiry, Patrick Fross, Hemkumar Srinivas, Thomas Pfeifer, Robert Moshammer, Anne Harth

**Affiliations:** 1 Max Planck Institute for Nuclear Physics, Saupfercheckweg 1, 69117 Heidelberg, Germany; 2 Center for Optical Technologies, Aalen University, Anton Huber Straße 1, 73430 Aalen, Germany

**Keywords:** Optics and photonics, Physics

## Abstract

For photo-dissociation of a single hydrogen molecule ($H_2$) with combined XUV and IR laser pulses, we demonstrate optical control of the emission direction of the photoelectron with respect to the outgoing neutral fragment (the H-atom). Depending on the relative delay between the two laser fields, adjustable with sub-femtosecond time resolution, the photoelectron is emitted into the same hemisphere as the H-atom or opposite. This emission asymmetry is a result of entanglement of the two-electron final-state involving the spatially separated bound and emitted electron.

## Introduction

Attosecond physics has developed into a central field that enables research into ultrafast coherent electron dynamics in matter, e.g. triggered by photoexcitation and photoionisation. A fundamental observation in this field is the emission behavior of photoelectrons after the dissociation of molecular hydrogen ($H_2$). Normally, the emission direction of a photoelectron is symmetric relative to the ejected neutral hydrogen atom, with no preferred direction, if the inversion symmetry (parity) of the electron wave function is maintained during the process. However, when the molecule is set in a superposition of states with opposite parities, an asymmetric electron emission pattern emerges^1^. This phenomenon emphasizes the interplay between molecular structure and electron dynamics. In order to thoroughly investigate the coherent dynamics triggered by photoionisation, in particular using the combination of attosecond laser pulses in the extreme ultraviolet (XUV) and infrared (IR) pulses, a detailed analysis of the resulting entanglement of ions and photoelectrons is essential and provides insight into the fundamental processes of attosecond physics.

Theoretical work is in progress that investigates the ultrafast coherent control of entangled states, e.g.^2–7^. Koll et al.^8^ discussed experiments on controlling quantum entanglement between an ion and a photoelectron in attosecond pump-probe experiments measuring the ion kinetic energy via a velocity map imaging device, by tailoring the spectral properties of attosecond XUV laser pulses. The research demonstrates how the degree of entanglement—and consequently, the vibrational coherence of the ion—can be manipulated. In our experiment, we demonstrate the asymmetric emission in the bipartite system of photoelectron/ion by detecting them in coincidence while they are spatially separated and steer their relative emission direction using the delay between XUV and weak IR pulses.

This effect should not be confused with the localization of the bound electron on one of the nuclei in the *laboratory frame* using a spatially asymmetric strong laser field during dissociation as reported in^9–12^. In those experiments, only the proton (deuteron) is detected and its ejection direction with respect to the laser polarization is controlled by the phase of the light field irrespective of the direction of the ejected photoelectron. Our study advances this by measuring both photo-electrons and protons in *coincidence*, assessing the emission direction in the *molecular frame*. Moreover, in contrast e.g. to Sansone’s experiment, where the lab-frame asymmetry was analyzed under the condition that ions appear with large kinetic energies—this is indicative of a fragmentation mechanism that involves the *decay of doubly-excited states* in the molecule—our study focuses on *ground-state fragmentation* via coherent superpositions of mixed-parity states. This approach is applicable to experiments using weak IR fields that do not introduce lab-frame asymmetry, and we observed no lab-frame asymmetry for either photoelectrons or ions. Finally, in certain photoninduced molecular fragmentation scenarios, a preferential photoelectron emission direction in the molecular frame suggests entanglement^13^, though such identification is complex and M. Vrakking emphasizes that true entanglement detection necessitates coincident two-particle measurement, moving beyond traditional attosecond experiment protocols that measure photo-electrons or ions separately^14^. This asymmetric photoelectron emission direction is sensitive to a phase difference between different dissociative pathways coming from e.g. opposite parity states. So far, this effect has been observed by single-photon dissociative photo-ionization^13,15^.

Our work overcomes experimental challenges to *control entanglement* by manipulating the relative phases between the different pathways from outside, achieving control over molecular frame asymmetry at a sub-femtosecond scale. We demonstrate active steering of the asymmetric electron emission by using a *few-photon* interaction with different colors in combination with the control of their relative delay. This capability is the core achievement of our study.

We determine the emission asymmetry from the angular distribution of the photoelectron with respect to the ejected neutral hydrogen atom containing the bound electron after the *n*-photon dissociation of $H_2$:

1 $$\begin{aligned} H_2+n\gamma \;\rightarrow \; H^+\;+\;H \;+\;e^- \end{aligned}$$

by detecting the electron $e^-$ and proton $H^+$ in coincidence using a reaction microscope (REMI) apparatus^16^, where the neutral hydrogen atom *H* is ejected opposite to the direction of the proton. $\gamma$ stands for the respective photon energies and *n* is the number of interacting photons. The spectrometer consists of two position-sensitive detectors for electrons and ions. We can retrieve the 3D momentum components of the ion $H^+$ and the electron $e^-$ using time-of-flights and hit positions on the detectors (see supplementary for more details).

Our light source is a femtosecond laser with a central wavelength of 1030 nm (IR) and a pulse duration of 50 fs. The beam is split into two arms. The main part is used to produce a comb-like ultraviolet (XUV) spectrum of odd harmonics with energies up to 40 eV using the process called high-harmonic generation^17^. A very schematic representation of a part of such a comb-like spectrum is visualized in blue and labeled with “$q+2$”, “*q*” and “$q-2$” in Fig. 2. The equally-separated maxima have an energy difference, which is twice the IR photon energy (2 $\times$ 1.2 eV). The remaining weaker part is delayed in time and overlapped spatially and temporally with the XUV pulses. Since any phase jitter between the XUV and IR pulses is critical for the experimential results, the beam line can be actively stabilized^18^. The basic experimental principles are similar to^19^. Both pulses interact with a gas jet of H$_2$ in the REMI.

**Figure 1:**
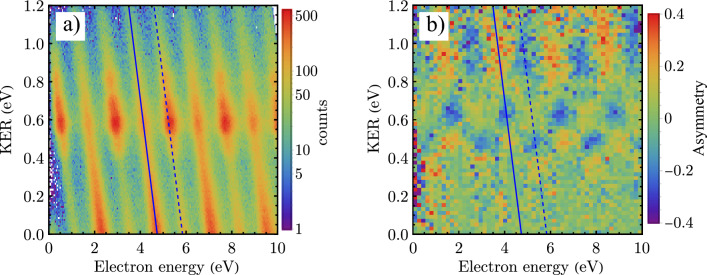
Interaction of XUV and IR photons with a hydrogen molecule: energy sharing spectrograms and asymmetry histograms. (**a**), Joint energy spectrum (JES) for the dissociative ionization of H$_2$. One example of an “odd band” (OB) is indicated with a diagonal solid line. This specific OB is a result of the absorption of XUV photons with an energy of 22.8 eV. The absorption of XUV + IR photons results in “even bands” (EB). An example of such an EB is marked with a diagonal dashed line. The enhancement of the signal at a KER of 0.6 eV is due to the absorption of an IR photon by the molecular ion (bond softening). (**b**) The asymmetry parameter in the case of dissociation with XUV and IR photons. The asymmetry is non-zero in the region between KER 0.35 eV to 1.2 eV. The two diagonal lines indicate the position of the marked OB and EB from (**a**) and serve as a guide.

The main process is single-ionization of H$_2$ into the molecular ion ground state (1$s\sigma _g$); only a small fraction of ionization events lead to dissociation for photons with energies higher than the dissociation limit of H$_2$ (I$_d$ = 18.1 eV). As described above, with the REMI we are able to obtain the energy and momentum (**K**) of both—the proton and the electron—for each dissociation event in coincidence. The energy of the not detected neutral hydrogen atom is reconstructed using momentum conservation: **K**$_{H}+$**K**$_{H^+}+$**K**$_{e^-}=0$. After dissociation, unlike atoms, the energy of the incoming photon can be distributed among electronic and nuclear degrees of freedom:

2 $$\begin{aligned} \overbrace{E_{H^+}+E_H}^\text {KER}+E_{e^-}= E_{\gamma }-I_d. \end{aligned}$$

The sum of the energy of the proton and hydrogen atom ($$E_{H^+}+E_H$$) is referred to as the kinetic energy release (KER). In order to visualize the distribution of the absorbed photon energy between nuclei and the electron, we plot the KER versus the electron energy ($$E_{e^-})$$ in a 2D histogram [joint energy spectrum (JES)]. Figure 1a shows a JES for photodissociation of H$$_2$$ with a combination of XUV and IR pulses in a KER region from 0 to 1.2 eV.

Due to energy conservation, most events appear on diagonal lines with a slope of − 1. Two types of such lines exist. We call them “odd bands” (OBs), mainly caused by single-XUV-photon absorption and “even bands” (EBs) caused by XUV-IR two-photon absorption. For example, absorption of XUV photons with an energy of 25.2 eV results in a total energy of 22.8–18.1 eV= 4.7 eV shared between electron and nuclei resulting in events on an OB highlighted by the diagonal solid line (Fig. 1a). A combination of XUV and IR photons results in an EB marked with a diagonal dashed line. The enhancement of the dissociation signal at a KER of around 0.6 eV is due to a process known as bond softening^20^.

**Figure 2:**
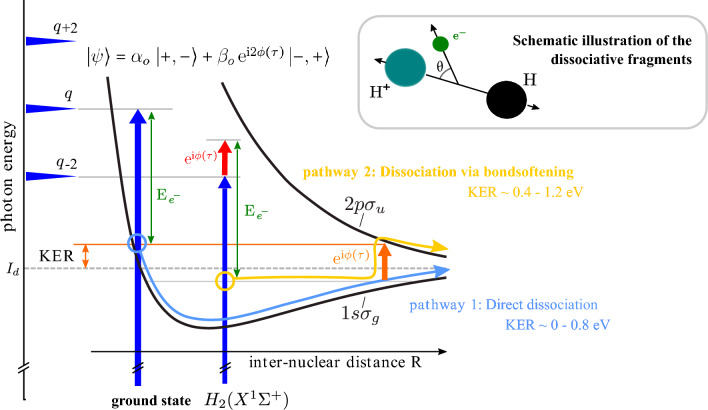
Schematic illustration of two dissociation channels of H$_2$ with a combination of XUV (blue arrows) and IR (red and orange arrows) photons. The ground state of H$_2$ ($X^1\Sigma ^+_g$) is a well defined state denoted with respect to its parity $|+,+\rangle$. The ground state ($1s\sigma _g$) and the first excited state ($2p\sigma _u$) of the molecular ion (H$_2^+$) are shown with black curves. The dissociation limit ($I_d$) is shown with the horizontal gray dashed line. Two indistinguishable dissociation pathways 1 (light blue curve, belonging to ground-state dissociation) and 2 (yellow curve belonging to bond softening) add up coherently to the same final state, namely the same photoelectron energy (E$_{e^-}$, green arrows) and KER. The inset shows the schematic illustration of the dissociative fragments: electron, proton and neutral hydrogen atom. $\theta$ is the emission angle of the photoelectron with respect to the H$^+$ emission direction.

In order to quantify an asymmetric electron emission, we define an asymmetry parameter *A*:

3 $$\begin{aligned} A=\frac{N_{\theta<90}-N_{\theta>90}}{N_{\theta <90}+N_{\theta >90}}, \end{aligned}$$

with $\theta$ being the electron emission angle with respect to the ejected proton as shown in the small box in Fig. 2. $N_{\theta <90}$ and $N_{\theta >90}$ are the numbers of events where the electron and proton are emitted in the same and the opposite hemisphere, respectively. A plot of the asymmetry parameter *A* for the events in Fig. 1a is shown in Fig. 1b. In a KER region from 0.4 to 1 eV, we clearly observe that the electron has a preferential direction with respect to the emitted proton (*A* is non-zero) for all electron energies. Additionally, OBs and EBs show different trends!

We like to note that the asymmetry A (see Eq. (3) and Fig. 1b) becomes visible only in the molecular frame or, in other words, when the relative emission-angle between electron and proton is plotted. When we analyze the proton emission in the lab-frame, without taking the electron data into account, we do not observe any asymmetry at all. This is not surprising because the acting light-field does not exhibit any asymmetry as well. It consists of an IR pulse with many cycles and an XUV field of odd harmonics where the individual XUV pulses appear in sync at each half-cycle of the IR-field. A clear lab-frame asymmetry in the proton emission can be measured^12^ if XUV pulses containing both odd and even harmonics are used. In this case, the symmetry of the light-field is broken because the XUV pulses appear at every second half-cycle of the IR-field, at instants when the electric field of the IR pulse is pointing into the same direction.

The energy-dependent asymmetry in 1b) is most pronounced in the region where two dissociation contributions overlap: the ground-state dissociation (contribution 1) and the bond softening (contribution 2). With the presence of many distinct XUV photon energies, several quantum paths within the two contributions exist and will interfere. However, only one pathway for each contribution is represented exemplarily in Fig. 2.

In case of OBs (see Supplementary material for EBs), in pathway 1 (light blue curve), an XUV photon with an energy $\gamma _q=q\,\hbar \omega$ (where $\omega =$1.2 eV is the photon energy of the laser radiation and *q* labels a maximum in the XUV spectrum) higher than $I_d$ can lead to dissociation along the 1s$\sigma _g$ state resulting in KERs < 2eV. This pathway leads to an electron energy of $E_{e^-}=\gamma _q-I_d -$ KER; the parity of the bound and the continuum electron becomes gerade $|+\rangle$ and ungerade $|-\rangle$, respectively.

In pathway 2 (yellow curve), the molecule is first ionized with the next lower harmonic photon ($\gamma _{q-2}= (q-2)\,\hbar \omega$), to a vibrational level of the ground state ($1s \sigma _g$) of the molecular ion. This process happens in the presence of a weak IR probe field where the photoelectron absorbs an IR photon instantaneously leading to a photoelectron energy of $E_{e^-}=\gamma _{q-2}+\hbar \omega -E_b$, where $E_b$ is the energy of the bound vibrational level. The symmetry of the emitted photoelectron after absorption of one IR photon becomes gerade $|+\rangle$. Further, the molecular ion, containing the bound electron, absorbs at a higher inter-nuclear distance R an additional IR photon leading the molecule to dissociate along the repulsive $2p\sigma _u$ ionic state. Thus, the bound electron is left with an ungerade symmetry $|-\rangle$.

The condition for quantum interference is fulfilled, when the total energy of both, the electron and the KER, are the same for both contributions. Hence, the final wave function can be written as a coherent superposition

4 $$\begin{aligned} |\psi \rangle = \alpha _o |+,-\rangle +\beta _o \textrm{e}^{i2\phi (\tau ) } |-,+\rangle \end{aligned}$$

where $\alpha _o$ and $\beta _o$ are complex probability amplitudes. Here we use the notation:

$$\begin{aligned} |\textsc {parity} \text { of the } \textsc {bound} \text { electron}, \textsc {parity} \text { of the } \textsc {free} \text { electron}\rangle . \end{aligned}$$

$\phi (\tau )$ is an XUV-IR pulse time-delay dependent phase shift. The vibrational nuclear wave-function can be omitted in the final wave function, since it is the same for both contributions for the same KER and always of gerade symmetry in case of H$_2$. Eq. (4) resembles the well-known Bell states: $|\psi _B^{\pm } \rangle = \frac{1}{\sqrt{2}}( |+,-\rangle \pm |-,+\rangle )$. And indeed, the observation of an asymmetric emission direction of the photoelectron with respect to its ion indicates that the two electrons—the photoelectron and the still bound electron—originating from the $H_2$ molecule are entangled with respect to parity^13^.

**Figure 3:**
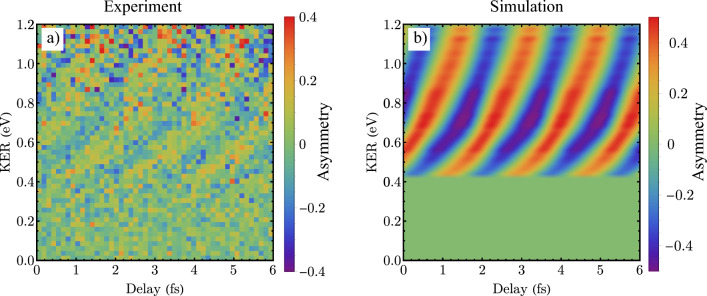
Time-dependent asymmetry parameter for OB: experiment vs. simulation. The asymmetry parameter *A* as a function of the time-delay between XUV and IR pulses. Comparison between the experiment (**a**) and simulation based on the WKB approximation (**b**). For the simulation, we plot Eq. (6) with retrieved $\alpha _o$ and $\beta _o$.

Since our experimental design allows to vary the phase $\phi (\tau )$ by changing the time delay between the XUV and IR pulses, we are able to investigate a delay dependent asymmetry parameter *A*. In order to show the time-dependence, we add all the OBs together and subtract the time-averaged asymmetry (see Supplementary material). The resulting asymmetry is plotted as a function of time-delay in Fig. 3a). The oscillation of the asymmetry parameter *A* as a function of the delay with a periodicity of 1.7 fs at a given KER demonstrates the sub-femtosecond laser control of the phase $\phi$ of the entangled states of Eq. (4).

**Figure 4:**
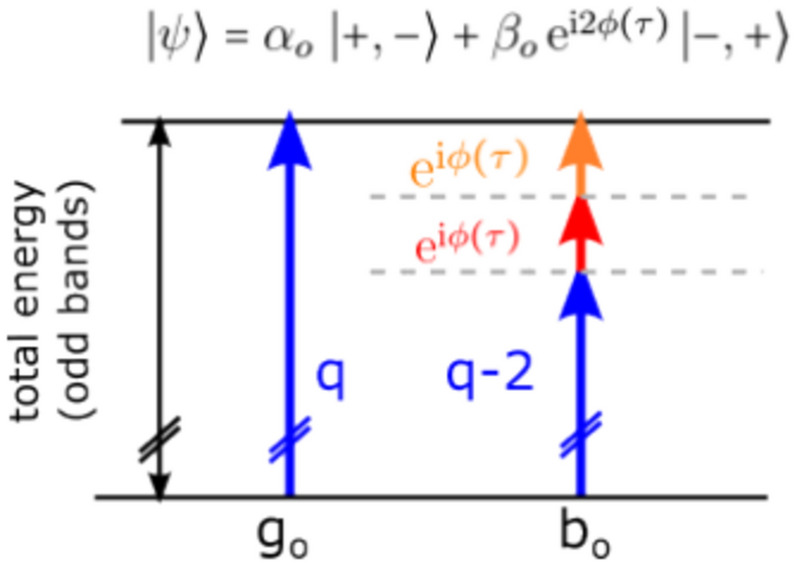
Schematic illustration of the generation of Bell-like states using a combination of high-(blue arrows) and low-frequency photons (red and orange arrows) from the ground state of molecular hydrogen. The distribution of photons among the different excitation pathways determines the generation of a specific Bell-like state.

Identifying the main contributing pathways allows us to model and simulate the time- and KER-dependent asymmetry. Two of three dominant pathways leading to OBs are illustrated in Fig. 4. We discuss the meaning of the third path in the supplementary and safely ignore this path here for simplicity. Figure 4 shows two energy states, which represents the initial state, the H$_2$ molecular ground state, and the final state, which includes the energy of the KER and the kinetic energy of the photoelectron. Both contributions are present: pathway 1 (or $g_{o}$) belongs to the ground state dissociation and pathway 2 (or $b_{o}$) to bond-softening dissociation. The color code in this Fig. 4 indicates the different function: the blue arrows illustrate two neighbouring XUV photons “*q*” and “$q-2$” that lead to photoionisation as explained in Fig. 2, the red arrow indicates that one IR photon is absorbed by the freed photoelectron after photoionisation and the orange arrow indicates the IR photon absorbed by the molecular ion, see Fig. 2. Identifying these paths, allows to express the complex amplitudes in Eq. (4) as $\alpha _o=g_{o}$ and $\beta _o=b_{o}\mathrm {e^{-\textrm{i}2\phi }}$ (see supplement) and Eq. (3) can be rewritten in terms of the new coefficients by using an appropriate base according to^13^:

5 $$\begin{aligned} A=\frac{-2|g_{o}||b_{o}| \text {cos}\big (\Delta \phi _{go,bo}-2\phi (\tau )\big )}{|g_{o}|^2+|b_{o}|^2}, \end{aligned}$$

with $\Delta \phi _{go,bo}=\text {arg}[g_o]-\text {arg}[b_o]$. This shows that the asymmetry parameter not only depends on amplitudes and the relative phase shifts between the different paths, but also on the time-delay which can be controlled in our experiment. The phase terms can be obtained by perturbation theory and the Wentzel–Kramers–Brillouin (WKB) approximation. The amplitudes are retrieved directly from the experimental data (see supplementary material). The intensity of the NIR field controls the ratio of the relative amplitudes. At higher NIR field strengths, additional (multi-photon) paths could contribute to the signals, opening additional routes for control. The experimental results are very well reproduced (compare Fig. 3a,b) with this model and the numerical simulation. This confirms that the presented time-controlled few-photon ionisation and dissociation process preserves the natural, ubiquitous entanglement of the two-electron system in the ground state of the $H_2$ molecule.

In conclusion, we have presented a proof-of-principle demonstration of a sub-femtosecond control of the phase between entangled states in molecular hydrogen by few-photon dissociation and coincident detection of all participating particles. It will be interesting to apply this scheme to larger molecules or even solids, and thereby test quantum-dynamical theories describing effective decoherence effects arising e.g. due to coupling to complex electronic or internuclear/phononic degrees of freedom. It should be noted that for suitably chosen quantum systems, even visible frequencies are sufficient to implement the same ultrafast control scheme.

## Methods

### Experimental methods

The output of a linearly-polarized fiber laser with a central wavelength of 1030 nm, a pulse energy of 1 mJ, and a pulse duration of 50 fs at a repetition rate of 50 kHz is divided into two beams with a 85/15 beam splitter and fed into an interferometer with a Mach–Zehnder configuration.

The main 85 percent (pump arm) is focused into an argon gas jet for high-harmonic generation (HHG) with a plano-convex lens with a focal length of 500 mm. A 200 $\upmu$m aluminum foil is used to filter the incoming infrared beam as well as the lower harmonics which results in the XUV spectrum shown schematically in Fig. S2c in the supplementary material. In the main text only a small region of the spectrum is shown for illustration purposes.

The remaining 15 percent (probe arm) is delayed in time by changing the length of the probe arm of the interferometer using a controllable piezoelectric linear translation stage. An iris is used to reduced the IR probe intensity to roughly $2\times 10^{11}$ $\text{ W/cm}^2$ in order to avoid higher-order photon-induced transitions during the experiment.

Both arms are recombined and focused using a grazing-incidence 2f–2f toroidal mirror into a supersonic gas jet of randomly-oriented cooled $H_2$ molecules inside a reaction microscope (cold target recoil ion momentum spectroscopy (COLTRIMS))^21^ where the background pressure is kept below $2\times 10^{-10}$ mbar. Both ions and electrons are guided towards position-sensitive detectors with the help of an electric field. A homogeneous magnetic field is also used to reach a $4\pi$-electron-detection efficiency. Momentum distributions of ions and electrons are obtained using the time of flight and the hit position on the detectors resulting in 3D momentum distributions. Three-particle momentum conservation is used to calculate the momentum vector of the missing H-atom. This way, the full kinematic information about the dissociation channels is obtained for each event. During the measurements, we simultaneously monitor the two-particle break-up of $H_2$ into a molecular ion $H_2^+$ and a photoelectron. From this, we obtain in-situ information about the combined resolution of ions and electrons, which is in the order of 0.2 a.u. (atomic units) for ions and about 0.1 a.u. or better for electrons. These values have to be compared with typical total momenta of more than $p = 5\,$ a.u. for the emitted H-atom (or proton) in case of dissociation. From these numbers, we conclude that the momentum of the undetected H-atom is reconstructed with an uncertainty of $\Delta p < 0.5$ a.u. or, in other words, with a relative accuracy of better than 10% for $\Delta p/p$. For an overview of the setup, see Fig. S1 in the supplementary material.

### Supplementary Information

Supplementary Information.

## Data Availability

Source data are provided in this paper. All other data that support the findings of this paper are available from the corresponding authors upon request.
