# Supplementary material for: Emission control of entangled electrons in photoionisation of a hydrogen molecule
Source: Sci Rep. 2024 Aug 23;14:19630. doi: 10.1038/s41598-024-67465-0 (PMC11343767; doi:10.1038/s41598-024-67465-0)
Supplement: Supplementary file 1 — Supplementary Information. [file 41598_2024_67465_MOESM1_ESM.pdf]

supplementary material for
Emission control of entangled electrons in photoionisation of a hydrogen molecule

Farshad Shobeiry[*,1], Patrick Fross[1], Hemkumar Srinivas[1], Thomas Pfeifer[*,1], Robert Moshammer[*,1], Anne Harth[*,1,2]

1 Max Planck Institute for Nuclear Physics, Saupfercheckweg 1, 69117 Heidelberg
2 Center for Optical Technologies, Aalen University, Anton Huber Straße 1, 73430 Aalen

[*]Corresponding authors. Email: shobeiry@mpi-hd.mpg.de, anne.harth@hs-aalen.de, thomas.pfeifer@mpi-hd.mpg.de, r.moshammer@mpi-hd.mpg.de.

1 Experimental setup

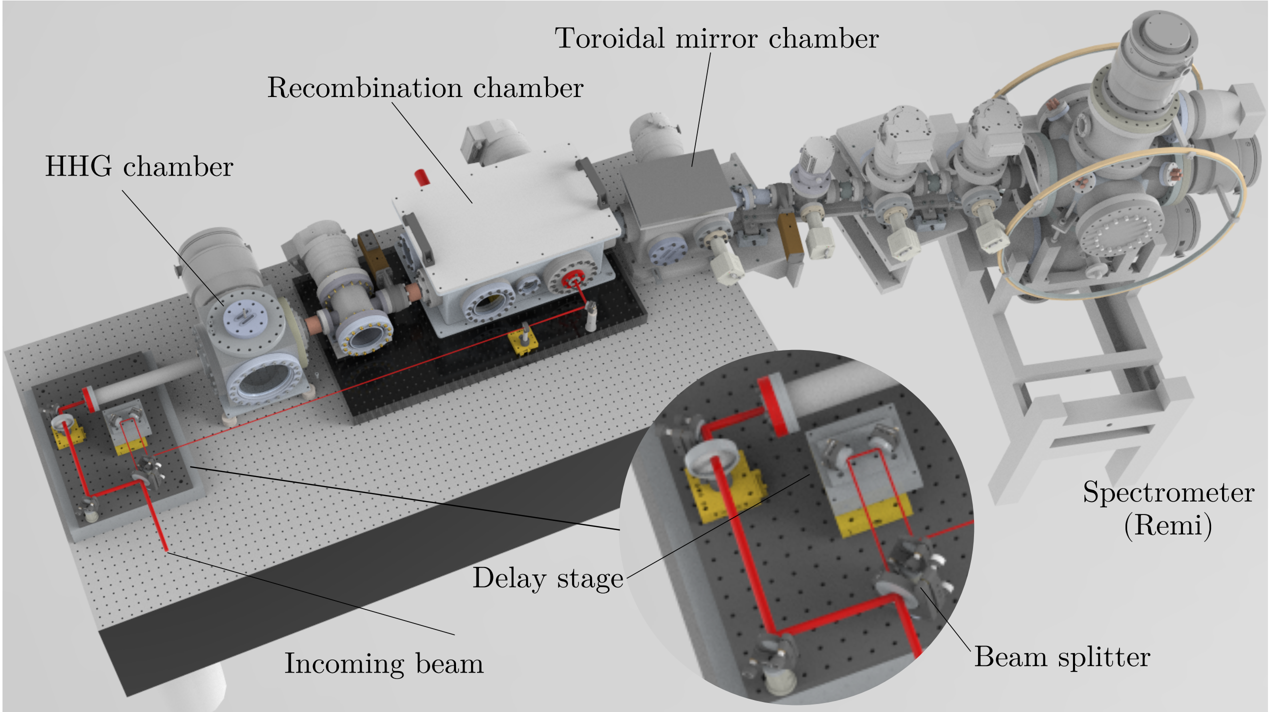

Fig. S 1: Overview of the attosecond beamline in Heidelberg.

The measurement was performed with the attosecond beamline (Fig. S1) at the Max Planck institute for nuclear physics in Heidelberg. The incoming laser beam is divided into two beams using an 85/15 beam splitter. The main arm (85 percent of the beam) is focused into a gas jet for high-harmonic generation in the HHG chamber. The remaining 15 percent (probe arm) is delayed in time by changing the optical path length of the probe arm using a controllable linear translation state. The two arms are recombined in a recombination chamber and focused with a toroidal mirror into the reaction microscope (REMI) (*1*). The interferometer is described in the Methods section of the main text.

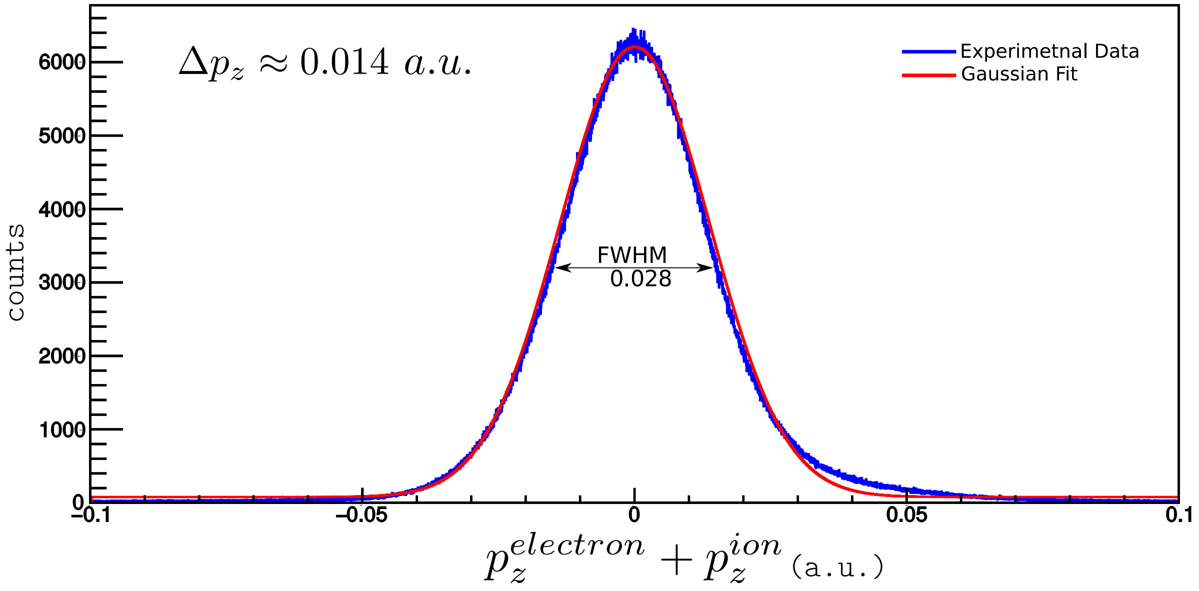

Fig. S 2: Sum of the longitudinal momentum components of electrons (e^-) and ions (H_2^+) in the single ionization of H_2. Figure taken from (*2*).

1.1 Momentum resolution of the REMI

Due to its geometry, the reaction microscope has a slightly different momentum resolution for different momentum components. The maximum resolution is along the spectrometer axis (longitudinal axis, z component). Fig. S2 and Fig. S3 show the sum of ion-electron (H_2^+-electron coincidence) momenta for the single-ionization channel in H_2. The width of the distributions is the measure of the momentum resolution. A dissociation event is determined by detecting an electron (e^-) and a proton (H^+) in coincidence. We recorded the data with a rate of 15 protons per second and detected in total over 2.1 millions H^+ events in coincidence with electrons.

1.2 Reconstruction of the molecular frame

The detected recoil direction is the same as the dissociation direction at the moment of the interaction between the photon with the molecule based on the axial recoil ap-

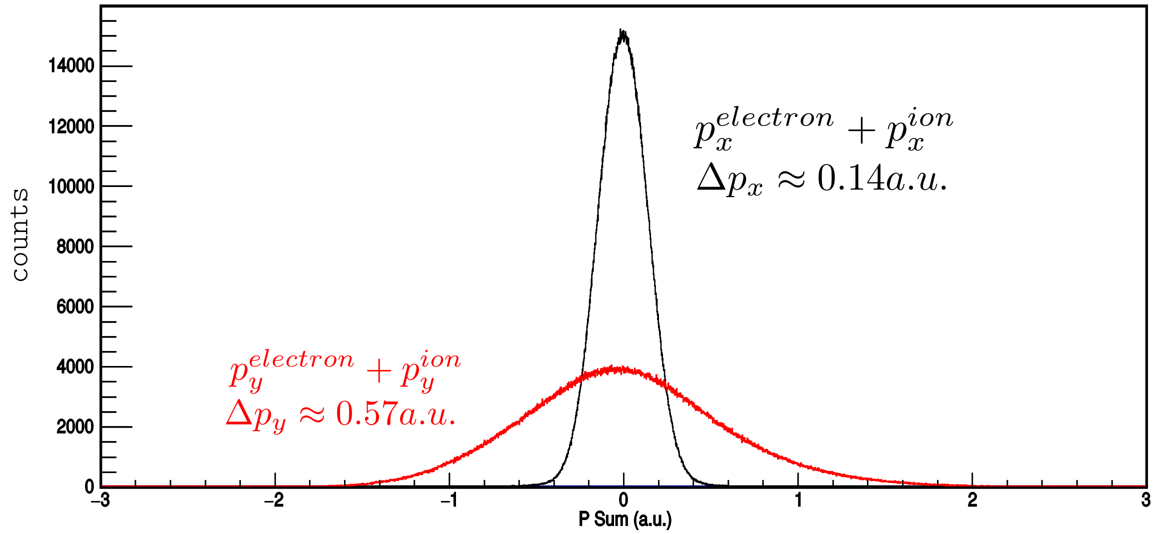

Fig. S 3: Momentum sum for the two transversal components of electrons (e^-) and ions (H_2^+) in the single ionization of H_2. Figure taken from (*2*).

proximation (*3*). Initially, all detected fragments are in the laboratory frame (LF) of reference. For photodissociation of H_2, the molecular frame (MF) momentum of the proton (see also Fig. S4) is given by:

$$\hat{\mathbf{p}}_{MF}^{H^+} = \hat{\mathbf{p}}_{LF}^{H^+} + \frac{1}{2}\hat{\mathbf{p}}_{LF}^{e^-}, \qquad (S.1)$$

where $\hat{\mathbf{p}}_{LF}^{H^+}$ and $\hat{\mathbf{p}}_{LF}^{e^-}$ are the retrieved momentum vectors in the LF for the proton and electron, respectively. The neutral fragment of the dissociation (H) is not detected during our measurement. However, using momentum conservation, we can reconstruct the momentum vectors of H in post-analysis. The momentum of the incoming photon is neglected, as it is much smaller than the momenta of the dissociation fragments and the spectrometer momentum resolution. For instance, the momentum of a photon with the energy of 30 eV is only 0.008 a.u. (1 a.u.of momentum is equivalent to 1.995×10^{-24} kg.m.s^{-1}) which is smaller than the momentum resolution of our spectrometer.

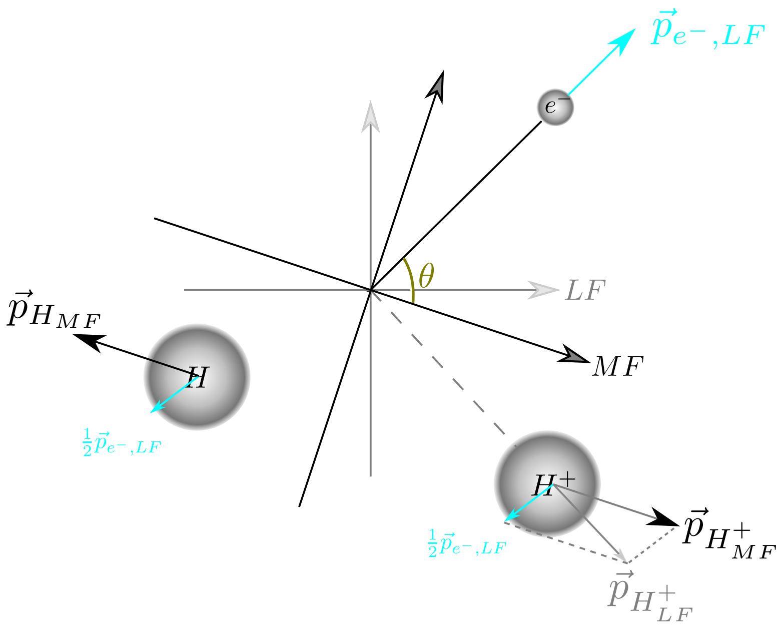

Fig. S 4: Schematic representation of the reconstruction of the molecular frame. Figure taken from (*2*).

2 Dissociation of H_2

Fig. S5 shows a typical time-of-flight (TOF) spectrum of the detected ions as a result of ionization of H_2 with XUV and IR photons. These data show that the dominant interaction product is the single-ionization of the molecule.

2.1 Interaction: XUV only

Upon the absorption of XUV photons with energies higher than the dissociation limit of H_2 (I_d=18.1 eV) the molecule can be ionized leading consecutively to the dissociation of the molecule. Fig. S6-A shows the schematic interaction of an XUV photon with H_2. The dissociation limit is marked with a horizontal dashed line at 18.1 eV. This photon belongs to the 21th harmonic of the XUV spectrum with an energy of 25.2 eV. A schematic representation of the high order harmonic spectrum is shown in Fig. S6-C. As a result, the molecule dissociates along the ionic state $1s\sigma_g$ (ground-state dissociation

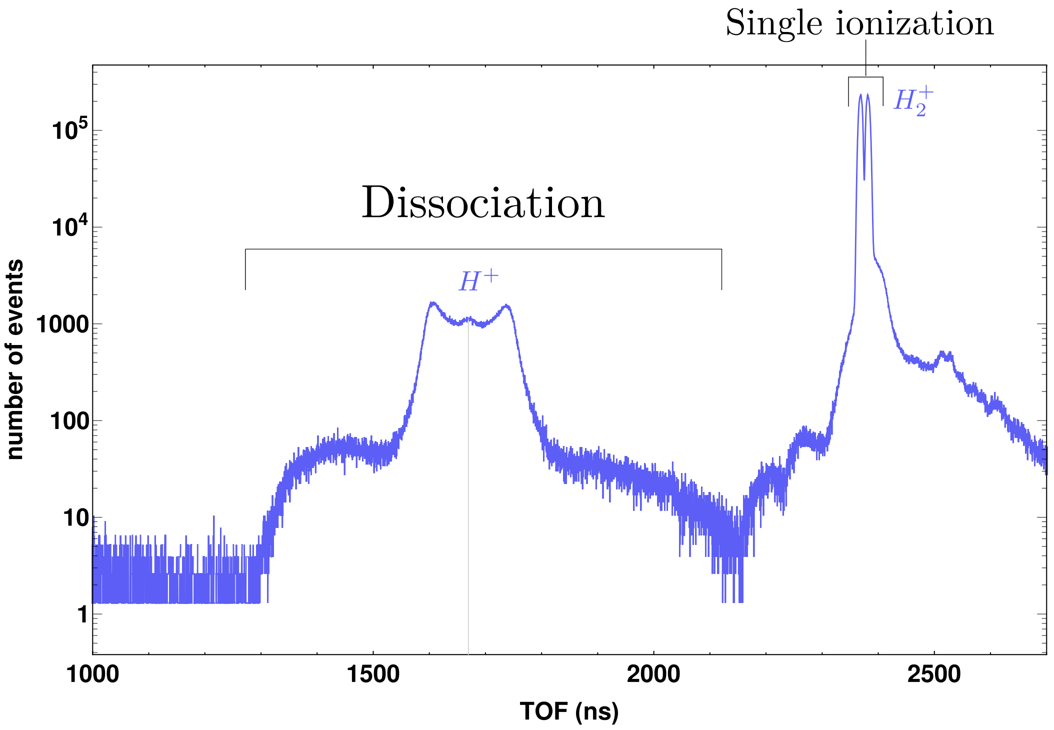

Fig. S 5: Time of flight spectrum of detected ions as a result of the interaction between a combination of XUV and IR pulses with H_2 molecules.

(GS)). GS is the dominant dissociative channel when applying XUV photon energies up to 27 eV (*4*). GS leaves the ionic fragment with an energy of less than 1 eV. The corresponding events from dissociation with harmonic 21st appear on a diagonal line marked with a solid blue line in the joint-energy spectrum (JES) histogram in Fig. S6-D. The other lines in Fig. S6-D correspond to other harmonics of the XUV spectrum. We call these lines "odd bands" (OBs) since an odd number of photons (in this case only one XUV photon) is absorbed. The projection of the marked band onto the KER axis is shown by the blue curve in Fig. S6-F.

2.2 Interaction: XUV + IR

The schematic interaction of a combination of XUV and weak IR photons with H_2 is shown in Fig. S6-B.

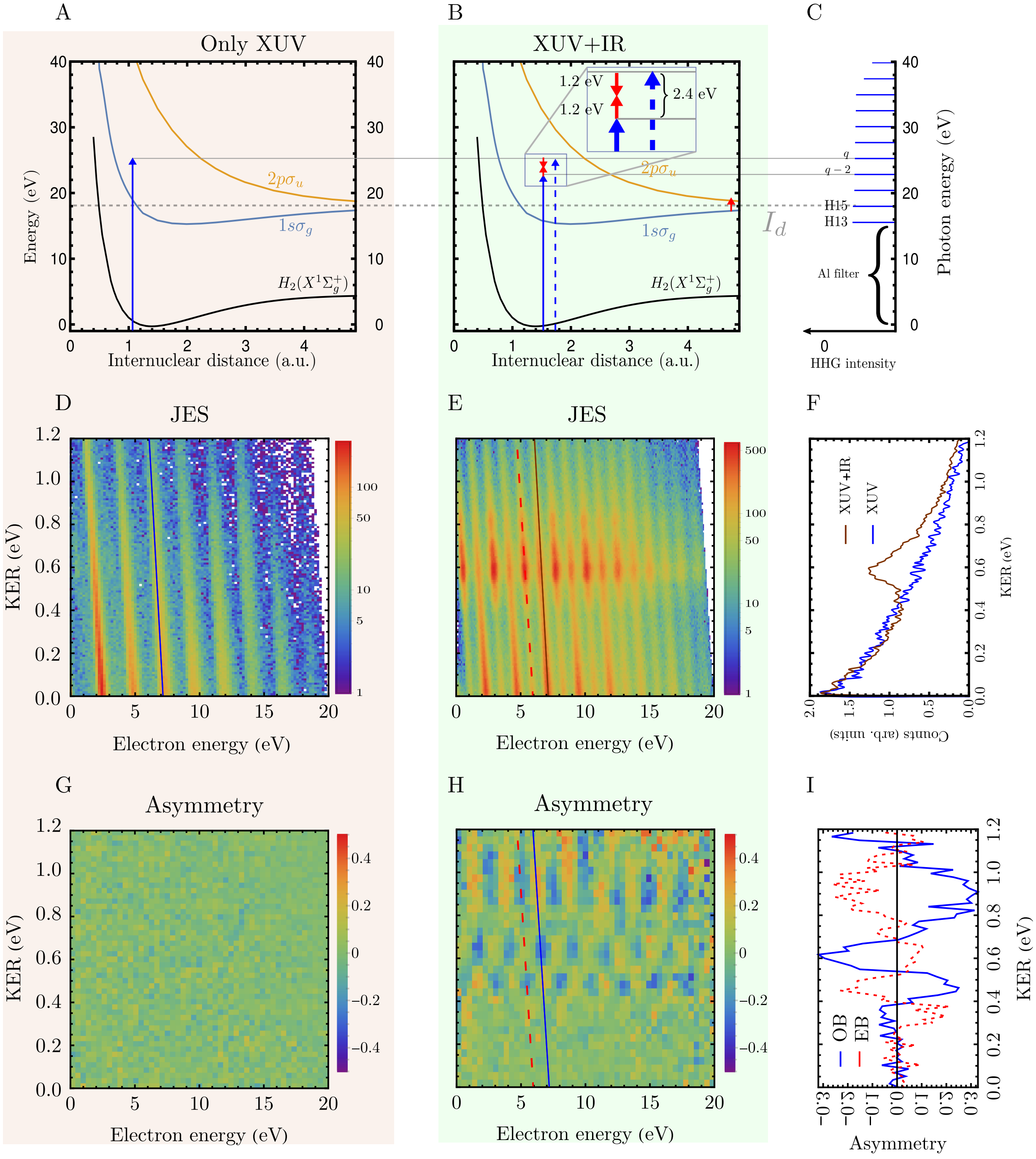

Fig. S 6: Dissociation of H_2: The relevant potential energy curves of H_2 are given in A and B. A: schematic interaction of the 21st harmonic only. B: schematic interaction of the XUV and IR photons. C: schematic spectrum of the XUV radiation resulting from high order harmonic generation. Lower part of the spectrum is filtered out by an aluminum filter. D: JES for the dissociation of H_2 with XUV light only. E: JES for the dissociation of H_2 with XUV+IR pulses. F: projection of the OB-21 created from XUV only (blue curve) and XUV+IR (brown curve) interaction onto the KER axis. G: asymmetry parameter A for XUV only. H: A for XUV+IR Pulses. I: A for EB 20 and OB 21.

In this case, one IR photon (red arrows) is absorbed or emitted (shown in the inset of panel B) by the photoelectron which results in events on diagonal lines (bands) between the OBs. One example is marked with a dashed red line in Fig. S6-E which we called an "even band" (EB) as a result of the absorption of e.g. an XUV photon from harmonic 19 plus an IR photon and/or the absorption of an XUV photon from harmonic 21 minus an IR photon.

In general, an additional IR photon can also be absorbed by the molecular ion due to bond softening at an internuclear distance (R) of around 5 a.u., where the 1sσ_g and 2pσ_u curves are energetically one IR photon apart (this process is indicated by the red arrow in Fig. S6-B at R ≈ 5 a.u.). This process results in an enhancement in the dissociation rate and is called bond softening (BS) dissociation.

The projection of the previously selected OB (this time marked with the brown solid line) in the case of the interaction with an XUV and an IR photon is shown by the brown curve in panel Fig. S6-F. We normalized the two curves at the KER around zero to emphasize the difference at a KER of around 0.6 eV. Note that, in this case, the region at KER ≈ 0.6 eV can be reached by the interaction with one XUV photon (from harmonic 21) only (GS) as well as by the interaction with one XUV photon from the next lower harmonic (harmonic 19) plus two IR photons (one absorbed by the photoelectron and the other by the molecular ion (BS)) or by one XUV photon from the same 21st harmonic followed by the emission of an IR photon by the photoelectron and absorption of another IR photon by the molecular ion (BS). (Note: The latter is a "third path" (see also Fig. S7), omitted in the main text for simplicity.)

2.3 Asymmetry parameter

The experimental results for the asymmetry parameter A are shown in Fig. S6-G and -H. Panel G shows that A is zero in the case of dissociation triggered only with XUV photons. This is a clear indication that the asymmetric photoelectron emission only takes place when different dissociation pathways (leading to the same final state) interfere. However, in Fig. S6-H we observe asymmetric photoelectron emission in the KER region where pathways contributing to BS and GS dissociation overlap (KER ≈ 0.6eV, see Fig. S6-F). An important feature in Fig. S6-H is that all OBs show the same trend. The same is true for EBs, all EBs show the same trend. Another feature is that the OBs and EBs are π out of phase due to the extra absorbed photon in the EBs. This feature is underlined in the projection of an odd and even band onto the KER axis in Fig. S6-I.

2.4 Pathways in even bands

In the discussion above, possible dissociation pathways according to the lowest order perturbation are described for the OBs. In the case of EBs (see e.g. Fig. S7), an XUV photon with an energy higher than I_d leads to dissociation through 1sσ_g with KERs<2 eV. The photoelectron emits an IR photon leaving the electron energy $E_e = \gamma_q - I_d - KER - \hbar\omega$ and the parity of the bound and continuum electron becomes gerade $|+\rangle$. For the pathway including bond softening, the molecule is ionized with the next lower harmonic into the ground state of the molecular ion 1sσ_g. The photoelectron energy is in this case $E_e = \gamma_{q-2} - E_b$ with E_b being the energy of the bound vibrational level in the ground state of the molecular ion. The parity of the photoelectron is ungerade $|-\rangle$. The molecular ion which contains the bound electron, absorbs an IR photon which promotes the molecular ion to the repulsive 2pσ_g curve. The molecule eventually dissociates, leaving the bound electron with ungerade parity $|-\rangle$.

2.5 Electron emission asymmetry

In this section, we establish a connection between the asymmetry parameter A (main text Eq. 3) and the coherent superposition of the dissociative pathways.

Since we have more than one pathway leading to the final state and these pathways are of two different natures - namely: ground-state (GS) dissociation and bond softening (BS) dissociation - we write the final state in a general manner in the form:

$$|\psi_{ob}\rangle = \boldsymbol{c}_{gs}\,|+,-\rangle + \boldsymbol{c}_{bs}\,|-,+\rangle\,, \tag{S.2}$$

for OBs and

$$|\psi_{eb}\rangle = \boldsymbol{c}_{gs}\,|+,+\rangle + \boldsymbol{c}_{bs}\,|-,-\rangle\,, \tag{S.3}$$

for EBs, where $\boldsymbol{c}_{gs}$ and $\boldsymbol{c}_{bs}$ are complex numbers. Both, the amplitudes and phases are relevant.

The superposition of molecular orbitals with different parities leads to the localization of a **bound electron** either on the left or right side of the nucleus. A similar spatial asymmetry occurs for a **photoelectron** placed in a superposition of spherical harmonics with either positive $|+_e\rangle$ or negative $|-_e\rangle$ parities (see (5)).

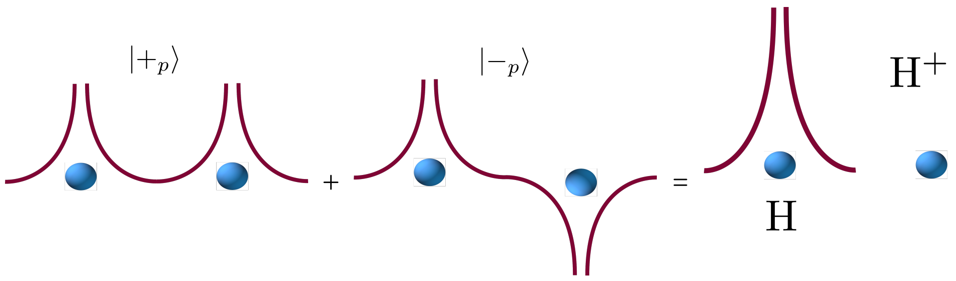

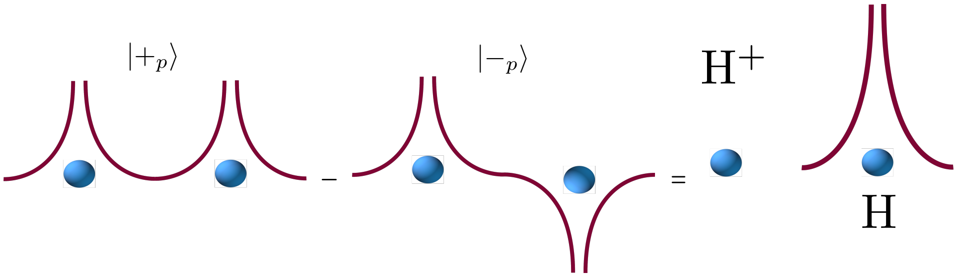

As a result, we can now define a left-right basis as follows:

$$\left|H^+_{right}\right\rangle = |+_p\rangle + |-_p\rangle\,,$$

$$\left|H^+_{left}\right\rangle = |+_p\rangle - |-_p\rangle\,,$$

$$\left|e^-_{right}\right\rangle = |+_e\rangle + |-_e\rangle\,,$$

$$\left|e^-_{left}\right\rangle = |+_e\rangle - |-_e\rangle\,.$$

This leads to the following four electron and proton combinations. Here exists two cases for both - the electron and the proton - being emitted in the same direction (and vice versa).

$$\left|\psi^1_{\theta<90}\right\rangle = \left|H^+_{right}\right\rangle \otimes \left|e^-_{right}\right\rangle\,,$$

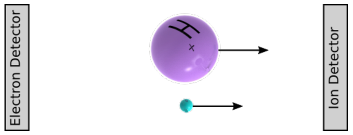

$$\left|\psi^2_{\theta<90}\right\rangle = \left|H^+_{left}\right\rangle \otimes \left|e^-_{left}\right\rangle\,,$$

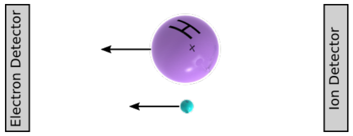

$$\left|\psi^1_{\theta>90}\right\rangle = \left|H^+_{right}\right\rangle \otimes \left|e^-_{left}\right\rangle\,,$$

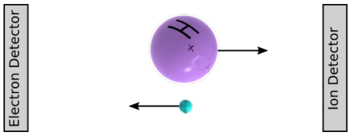

$$\left|\psi^2_{\theta>90}\right\rangle = \left|H^+_{left}\right\rangle \otimes \left|e^-_{right}\right\rangle\,,$$

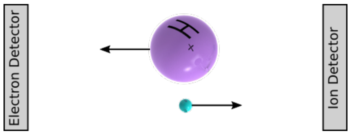

θ is the angle between the photoelectron and the ejected proton (see Fig. S4 and Fig. 2 in the main text). The transition coefficients c_{gs} and c_{bs} can be calculated by projecting

the final state (Eq.S.3, Eq.S3) onto different direction states using the above equations:

$$\begin{aligned}\left|\left\langle\psi^1_{\theta<90}\middle|\psi\right\rangle\right|^2 &= (c_{gs}+c_{bs})(c^*_{gs}+c^*_{bs}),\\ \left|\left\langle\psi^2_{\theta<90}\middle|\psi\right\rangle\right|^2 &= (-c_{gs}-c_{bs})(-c^*_{gs}-c^*_{bs}),\\ \left|\left\langle\psi^1_{\theta>90}\middle|\psi\right\rangle\right|^2 &= (-c_{gs}+c_{bs})(-c^*_{gs}+c^*_{bs}),\\ \left|\left\langle\psi^2_{\theta>90}\middle|\psi\right\rangle\right|^2 &= (c_{gs}-c_{bs})(c^*_{gs}-c^*_{bs}).\end{aligned} \tag{S.4}$$

We define the number of dissociation events as:

$$\begin{aligned}N_{\theta<90} &= \left|\left\langle\psi^1_{\theta<90}\middle|\psi_f\right\rangle\right|^2 + \left|\left\langle\psi^2_{\theta<90}\middle|\psi_f\right\rangle\right|^2,\\ N_{\theta>90} &= \left|\left\langle\psi^1_{\theta>90}\middle|\psi_f\right\rangle\right|^2 + \left|\left\langle\psi^2_{\theta>90}\middle|\psi_f\right\rangle\right|^2.\end{aligned} \tag{S.5}$$

Using now the definition of the asymmetry parameter A (Eq.3 of the main text), we can rewrite A as a function of $\boldsymbol{c}_{gs}$ and $\boldsymbol{c}_{bs}$:

$$\begin{aligned}A &= -\frac{2\mathrm{Re}[c_{gs}c^*_{bs}]}{|c_{gs}|^2+|c_{bs}|^2}\\ &= -\frac{2|c_{gs}||c_{bs}|\cos(\phi_{gs}-\phi_{bs})}{|c_{gs}|^2+|c_{bs}|^2},\end{aligned} \tag{S.6}$$

where $\phi_{gs,bs} = \arg[c_{gs}, c_{bs}]$.

3 Model based on perturbation theory and WKB approximation

In this section we introduce a model that supports that the origin of the time-dependent asymmetry lies in the interference of photoelectrons coming from GS and BS dissociation quantum pathways where at least two neighboring harmonics in the XUV spectrum are involved.

Many quantum pathways are involved in the experiment. However, for the GS and BS dissociation pathways, we consider only the lowest photon transitions due to vanishing intensities of both XUV and IR pulses. These lowest order pathways are shown

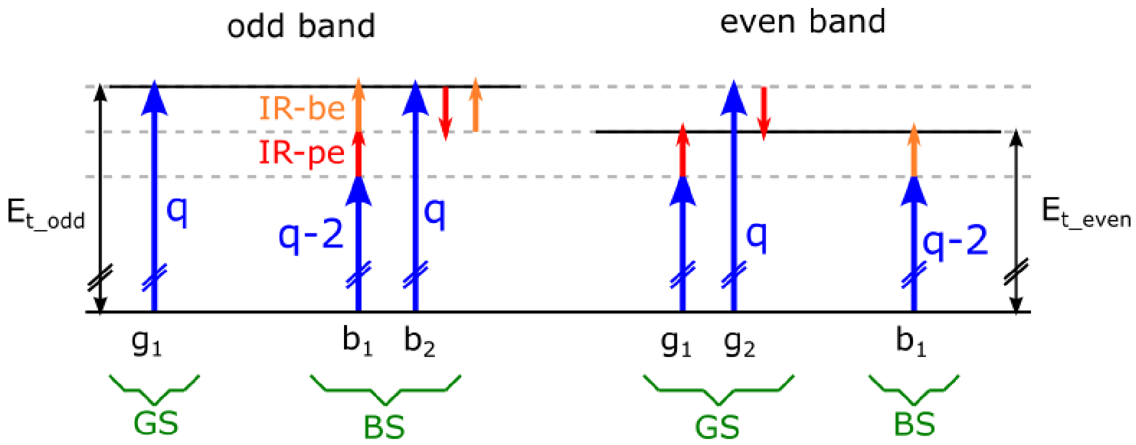

Fig. S 7: **Quantum pathways for odd and even bands**. The blue arrows represent the XUV photon with different discrete photon energies: e.g. $q-2$ is the next lower harmonic to q with an energy difference of two IR photons. The red arrow represents an IR photon absorbed or emitted by the photoelectron (IR-pe), while the orange one represents an IR photon absorbed by the bound electron (IR-be) leading to bond softening.

in Fig. S7. All these paths interfere and have a contribution to the final dissociation probability, and affect the asymmetry parameter A.

3.1 Odd bands

For now, we consider **odd bands** only. The following discussion is repeated for even bands in section 3.5. The complex coefficients are called g_1 for the GS pathway, b_1 and b_2 for the BS pathway according to Fig. S7. Eq.S.2 becomes:

$$|\psi\rangle = g_1 |+,-\rangle + (b_1 + b_2) |-,+\rangle . \tag{S.7}$$

Note that for pathway g_1 one photon and for pathways b_1 and b_2 three photons are involved. With this, the dissociation probability reads:

$$P_{odd} = |g_1 + b_1 + b_2|^2 . \tag{S.8}$$

The asymmetry parameter from Eq. S.6 becomes:

$$\begin{aligned} A_{odd} &= \frac{-(g_1(b_1+b_2)^* + (b_1+b_2)g_1^*)}{|b_1+b_2|^2 + |g_1|^2} \\ &= \frac{-2(|g_1||b_1|\cos(\Delta\phi_{g_1,b_1}) + |g_1||b_2|\cos(\Delta\phi_{g_1,b_2}))}{|g_1|^2 + |b_1|^2 + |b_2|^2 + 2|b_1||b_2|\cos(\Delta\phi_{b_1,b_2})}, \end{aligned} \tag{S.9}$$

with $\Delta\phi_{bi,bj} = \arg[b_i] - \arg[b_j]$ and $\Delta\phi_{gi,bj} = \arg[g_i] - \arg[b_j]$.

The complex coefficients correspond to the specific components of the dipole transition element from the vibronic ground state to the continua (6). Within the Franck-Condon (FC) approximation, the electronic and the nuclear components of the total wave function (Eq.S.7) can be separated $\psi(r,R) = \chi(R)\phi(r,R)$. We neglect the dependence of the electronic matrix elements on the nuclear position. This leads to the complex expressions:

$$g_1 = \underbrace{FC_{gs}\,\mathrm{e}^{\mathrm{i}\Theta_{gs}}}_{\substack{nuclear\\contribution}} \times \underbrace{M_{g1}^{(1)}}_{\substack{photo-e\\contribution}}, \tag{S.10}$$

$$b_1 = \underbrace{FC_{bs}\,\mathrm{e}^{\mathrm{i}\Theta_{bs}}}_{\substack{nuclear\\contribution}} \underbrace{\mathrm{e}^{\mathrm{i}\phi(\tau)}}_{\substack{bound-e\\contribution}} \times \underbrace{M_{b1}^{(2)}}_{\substack{photo-e\\contribution}}, \tag{S.11}$$

$$b_2 = \underbrace{FC_{bs}\,\mathrm{e}^{\mathrm{i}\Theta_{bs}}}_{\substack{nuclear\\contribution}} \underbrace{\mathrm{e}^{\mathrm{i}\phi(\tau)}}_{\substack{bound-e\\contribution}} \times \underbrace{M_{b2}^{(2)}}_{\substack{photo-e\\contribution}}, \tag{S.12}$$

where FC stands for a contribution that represents the nuclear part. The nuclear part has a phase $\Theta_{gs/bs}$. The contribution for the photoelectron is accounted for by the complex multi-photon matrix element $M^{(N)}$ where N is the number of involved photon transitions. Bond softening is a photo-induced process. In order to account for the phase of this field, the phase of the IR field $e^{i\phi(\tau)}$ is multiplied to the bond softening matrix element in Eq.S11 and S12.

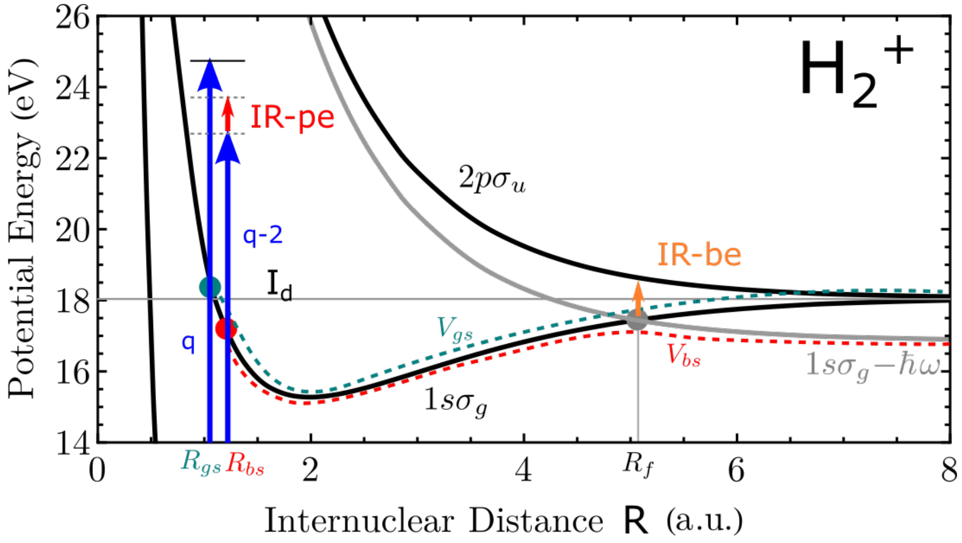

Fig. S 8: Schematic representation of the quantum paths g_1 and b_1 in the H_2 molecular potential curves. These two quantum paths are the most significant ones that lead to a time-dependent asymmetry in odd bands.

3.2 WKB-phase

The phases $\Theta_{gs/bs}$ account for the accumulated phases of the nuclei moving in the given potential energy curves V_{gs} and V_{bs}, see Fig. S8. The phases from the moving nuclei are estimated with the WKB Wentzel–Kramers–Brillouin (WKB) approximation:

$$\Theta_{gs} = \int_{R_{gs}}^{\infty} dR\sqrt{2\mu(KER + I_d - V_{gs}(R))}, \tag{S.13}$$

$$\Theta_{bs} = \int_{R_{bs}}^{\infty} dR\sqrt{2\mu(KER + I_d - V_{bs}(R))}, \tag{S.14}$$

where the integrand is the nuclear momentum with μ being the reduced mass of H_2^+.

Please note the different limits of the integrals. For ground-state dissociation, the phase Θ_{gs} is calculated by integrating from the internuclear distance R_{gs} to the limit of $R \to \infty$ where the molecule dissociates along the $1s\sigma_g$ curve with a KER of 0.6 eV. The phase of the pathway (Θ_{bs}) is obtained by the integral in equation S14 which has a different lower limit since the process starts with the ionization of the molecule leaving the ion in the bound ground state. The integration pathway follows the laser-dressed poten-

tial curve (dashed red curve (V_{bs})) at the limit of the adiabatic $1s\sigma_g$-$\hbar\omega$ curve obtained by diagonalizing the diabatic potential matrix (*7*). The width of the avoided crossings at R_f is obtained by taking into account the IR intensity during the measurement.

3.3 Phase of electric dipole matrix element

For this model approach, the electric dipole matrix elements, M, are approximated to be independent of R. According to the shown paths in Fig. S7, $M_{g1}^{(1)}$ describes a first-order perturbation process and $M_{b1}^{(2)}, M_{b2}^{(2)}$ describe second-order perturbation processes. Although we deal with a real two-electron system, the single active electron approximation can be used (*8*), since the interaction with the light only changes the electronic state of the photo-electron; while the bound electron remains in the ground state of the molecular ion.

The complex electric dipole matrix elements (assuming only linearly polarized light) and their asymptotic phases can be written as (*9*):

$$M_{g1}^{(1)}(k;R_{gs}) = E_{\Omega_q}\sum_{\ell}\mathcal{M}_{\ell,0}^{(1)}(k)Y_{\ell,0} \quad \text{with} \quad \arg[M_{g1}^{(1)}] = \phi_q^{XUV} - \frac{\pi}{2} - \frac{\pi\ell}{2}, \tag{S.15}$$

$$M_{b1}^{(2)}(k;R_{bs}) = E_{\Omega_{q-2}}E_{\omega}\sum_{\ell}\mathcal{M}_{\ell,0}^{(2)}(k)Y_{\ell,0} \quad \text{with} \quad \arg[M_{b1}^{(2)}] = \phi_{q-2}^{XUV} + \omega\tau - \frac{\pi\ell}{2}, \tag{S.16}$$

$$M_{b2}^{(2)}(k;R_{bs}) = E_{\Omega_q}E_{-\omega}\sum_{\ell}\mathcal{M}_{\ell,0}^{(2)}(k)Y_{\ell,0} \quad \text{with} \quad \arg[M_{b2}^{(2)}] = \phi_q^{XUV} - \omega\tau - \frac{\pi\ell}{2}. \tag{S.17}$$

Here, the atomic phase contribution including the Wigner and continuum-continuum couplings are neglected, since they are small compared to the atto-chirp (*10*). The final photoelectron momentum k and angular momentum ℓ are the same for all paths. $E_{\Omega/\omega}$ are the complex electric fields of the XUV and the IR pulses, $Y_{\ell,m}$ are the angular momentum and magnetic-quantum-number dependent spherical harmonics. $\mathcal{M}^{(N)}(k)$ is a complex expression that contains the radial part of the transition-matrix element. We can safely assume that the IR pulse $\arg[E_\omega] = \omega(t+\tau) + IR_{chirp}$ is not chirped and we

set $t = 0$: $\arg[E_\omega] = \omega\tau = \phi(\tau)$. With this, we write the phases of the complex amplitudes of the three quantum paths for odd bands as follows:

$$\arg[g_1] = \Theta_{gs} - \frac{\pi}{2} - \frac{\pi\ell}{2} + \phi_q^{XUV} \qquad = \Phi_{g1}, \tag{S.18}$$
$$\arg[b_1] = \Theta_{bs} - \frac{\pi\ell}{2} + \phi_{q-2}^{XUV} + 2\phi(\tau) \qquad = \Phi_{b1} + 2\phi(\tau), \tag{S.19}$$
$$\arg[b_2] = \Theta_{bs} - \frac{\pi\ell}{2} + \phi_q^{XUV} \qquad = \Phi_{b2}. \tag{S.20}$$

To obtain Eq.4 and 5 in the main text, we replace the complex coefficients in Eq. S.7 with the defined phases above and their corresponding magnitudes and get:

$$|\psi\rangle = |g_1|\mathrm{e}^{i\Phi_{g1}}\,|+,-\rangle + \left(|b_1|\mathrm{e}^{i(\Phi_{b1}+2\phi(\tau))} + |b_2|\mathrm{e}^{i\Phi_{b2}}\right)|-,+\rangle\,. \tag{S.21}$$

By setting $|g_1|\mathrm{e}^{i\Phi_{g1}} = \alpha_o$, $|b_1|\mathrm{e}^{i\Phi_{b1}} = \beta_o$, and neglecting pathway b_2, we get

$$|\psi\rangle = \alpha_o\,|+,-\rangle + \beta_o\mathrm{e}^{i2\phi(\tau)}\,|-,+\rangle\,. \tag{S.22}$$

We discuss this simplification in order to focus on the time-dependence asymmetry. However, path b_2 is needed in order to explain all observed structures in the experiment.

The asymmetry parameter (Eq. S.10) in this case becomes

$$A = \frac{-2|\alpha_o||\beta_o|\cos\left(\arg[\alpha_o] - \arg[\beta_o] - 2\phi(\tau)\right)}{|\alpha_o|^2 + |\beta_o|^2}. \tag{S.23}$$

In order to obtain an analytical expression for the asymmetry (Eq. S.9), we need to know the phase differences between the three paths:

$$\begin{aligned}
\Delta\phi_{g_1,b_1} &= \Delta\Theta - \frac{\pi}{2} + \Delta\phi_{q,q-2}^{XUV} - 2\phi(\tau),\\
\Delta\phi_{g_1,b_2} &= \Delta\Theta - \frac{\pi}{2},\\
\Delta\phi_{b_1,b_2} &= +\Delta\phi_{q-2,q}^{XUV} + 2\phi(\tau),
\end{aligned} \tag{S.24}$$

where $\Delta\phi_{i,j}^{XUV} = \phi_i^{XUV} - \phi_j^{XUV}$ is the chirp of the XUV pulse, and $\Delta\Theta = \Theta_{gs} - \Theta_{bs}$ is the phase difference resulting from the nuclear part (WKP phase) of the wave function. The

time dependent asymmetry parameter then reads:

$$A_{odd}(\tau) = \frac{-2|g_1||b_1|\sin\big(\Delta\Theta + \Delta\phi^{XUV}_{q,q-2} - 2\phi(\tau)\big) - 2|g_1||b_2|\sin(\Delta\Theta)}{|g_1|^2 + |b_1|^2 + |b_2|^2 + 2|b_1||b_2|\cos\big(\Delta\phi^{XUV}_{q,q-2} - \phi(\tau)\big)}, \quad \text{(S.25)}$$

and the time-averaged asymmetry parameter is not zero: $\langle A\rangle_\tau = \int d\tau A(\tau) \neq 0$.

It is worth discussing the role of the different considered quantum paths g_1, b_1,and b_2. If $|g_1|$ is zero, the asymmetry parameter is zero and the time-dependent population probability shows a similar form of a RABBITT-like experiment (*11, 10*). If $|b_1|$ is zero, the asymmetry parameter is not time dependent. This shows that, in order to see a time dependence in the experiment, two interfering quantum paths need to involve two neighboring XUV frequencies. If $|b_2|$ is zero, the asymmetry parameter is time dependent but the time average asymmetry parameter is zero. Only if all three paths are present, we obtain what the experiment shows:

- a τ-dependent asymmetry parameter $A(\tau)$
- and a non zero time average $\langle A\rangle = \int d\tau A(\tau) \neq 0$.

Therefore, at least these three lowest-order perturbation paths must be considered in order to model the experimental results. However, for the discussion in the main text regarding the origin of the time-dependent asymmetry, two paths are sufficient.

3.4 Experiment

3.4.1 Time-integrated OB asymmetry parameter and chirp of the XUV pulses

Fig. S9 shows the experimental time-averaged asymmetry parameter $\langle A\rangle$ as a function of KER for the first three odd bands (OBs). They correspond to harmonic 17th (OB1), 19th (OB2), and 21st (OB3). The black line is the theoretical curve based on the model

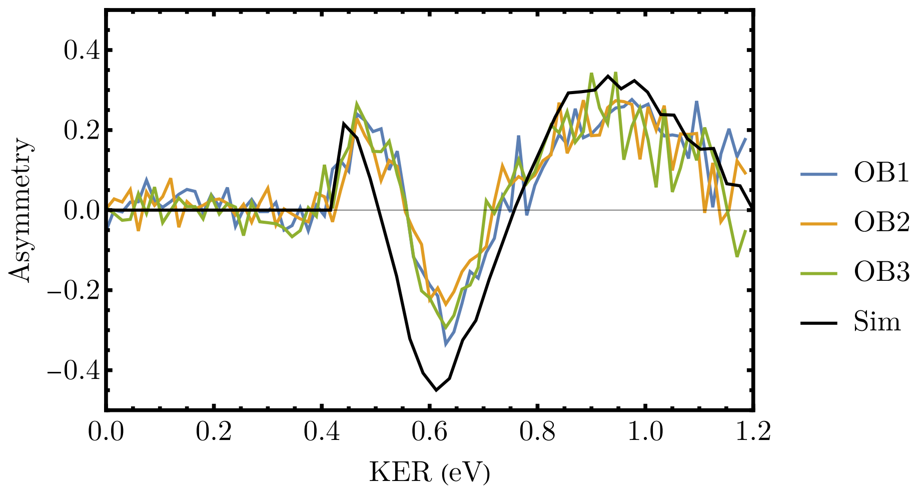

Fig. S 9: Experimental time-integrated odd-band asymmetry parameter $\langle A \rangle$ as a function of KER. Black curve shows the asymmetry parameter A based on Eq. S.25 with $|g_1| = \alpha_o$ and $|b1 + b2| = \beta_o$.

explained above. For the simulation we used the retrieved amplitudes for g_1, b_1, and b_2 explained in the following section 3.5. For $\phi(\tau) = \omega\tau$, we know the photon energy (1.2 eV) and therefore ω. Before plotting the experimental asymmetry parameter as a function of the time delay, we add all OBs together in order to improve the statistics. The bands are slightly shifted in time with respect to each other mainly due to the chirp of the XUV pulse. This chirp is a function of the photon energy. In order to obtain the XUV chrip, we perform a reference measurement on argon prior to the measurement on H_2. We use the photoelectron spectrum as a function of the time delay between XUV and IR pulses (also known as the RABBIT spectrum) to retrieve the chirp of the XUV pulse. Fig. S10 shows the relative phase of the different side-bands in the RABBIT spectrum.

3.4.2 Coefficients α and β for even and odd bands

We can retrieve the coefficients $\alpha_{o/e}$ and $\beta_{o/e}$ in Eq. 4 of the main text using the KER distribution. An example is shown with the blue curve in Fig. S11 for an OB (a) as

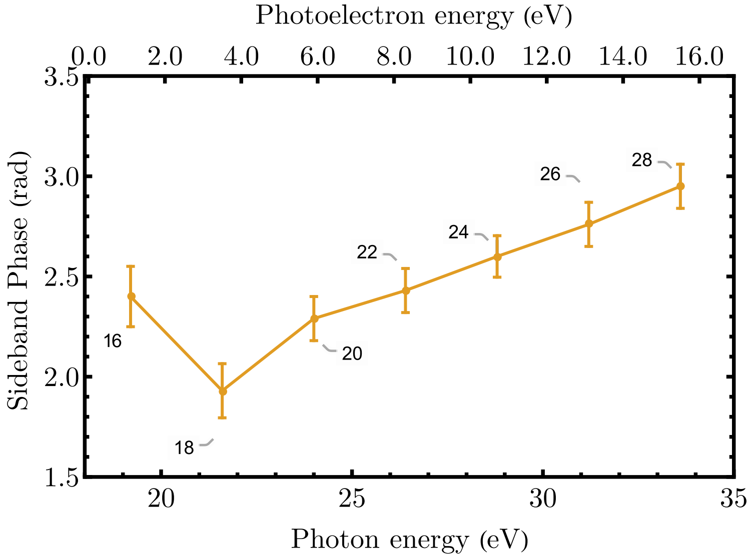

Fig. S 10: The chirp of the XUV pulses. Shown are the relative phases of the side-bands in photoionisation of argon using a combination of XUV and IR pulses. The figure is adapted from (*2*).

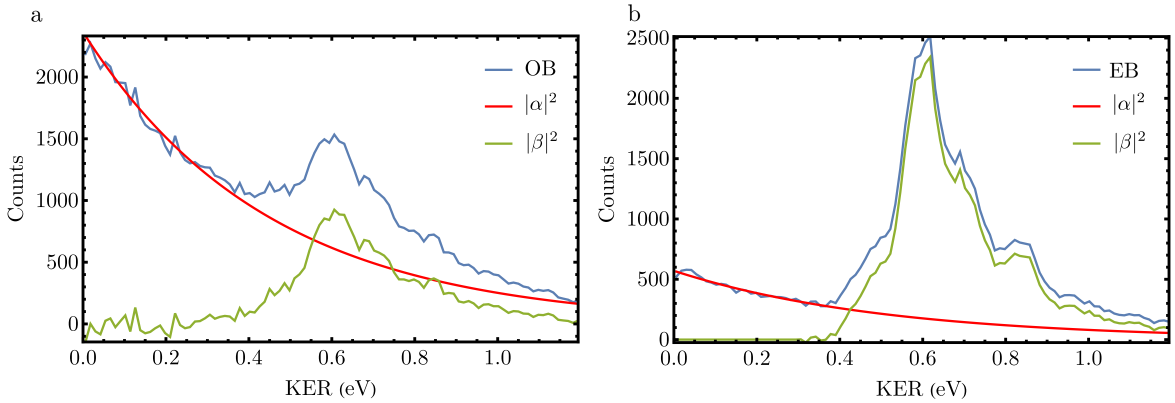

Fig. S 11: Extraction of α and β from the experimental data. a: The blue curve represents the KER distribution of the third OB (marked with a solid line in Fig. S6-E). b: The blue curve represents the KER distribution of the third EB (marked with a dashed line in Fig. S6-E). The red curve is and an exponential function fitted to the blue curve up to a KER of 0.35 eV giving us $|\alpha|^2$. The green curve is obtained by subtracting the red curve from the blue one to get $|\beta|^2$.

well as an EB (b). For the contribution of the ground state dissociation (α), we fit an exponential function of the form $f(x) = Ue^{-|u|x}$ to a KER region from 0 to 0.35 eV and obtain the exponential KER trend for the rest of the KER region up to 1.2 eV shown with the red curve in Fig S 11. The KER region up 0.35 eV contains only the contribution form GS dissociation. Then we set $|\alpha|^2 = f(x)$. The fit is then subtracted from the experimental data to obtain the contribution of the bond softening (β). In case of odd bands, according to Eq. S.7, we have $\beta_o = b_1 + b_2$ and we set $b_1 = b_2$. For EBs, according to Eq. S.26, $\alpha_e = g_1 + g_2$ and we set $g_1 = g_2$.

3.4.3 Time-dependent asymmetry parameter A for odd bands: Experiment and simulation

The final plots for the asymmetry parameter A for odd bands are shown in Fig. S12-A. Fig. 12-B shows the corresponding simulated case based on the WKB approximation, the model above and the retrieved α and β parameters. Fig. S12-C and -D shows the asymmetry parameter from -A and -B minus the mean time-averaged contribution $A(\tau) - \langle A(\tau)\rangle$ for both the experiment and theory. The experimental and simulated results show a good agreement.

3.5 Even-bands

In this final section, the same procedure as for odd bands is now repeated for even bands (EBs), where the total number photons absorbed in each pathway is even. According to the pathways for EBs shown in Fig. S7, Eq. S.2 becomes:

$$|\phi\rangle = (g_1 + g_2)\,|+,+\rangle + b_1\,|-,-\rangle\,. \qquad \text{(S.26)}$$

The population probability reads

$$P_{even} = |g_1 + g_2 + b_1|^2, \qquad \text{(S.27)}$$

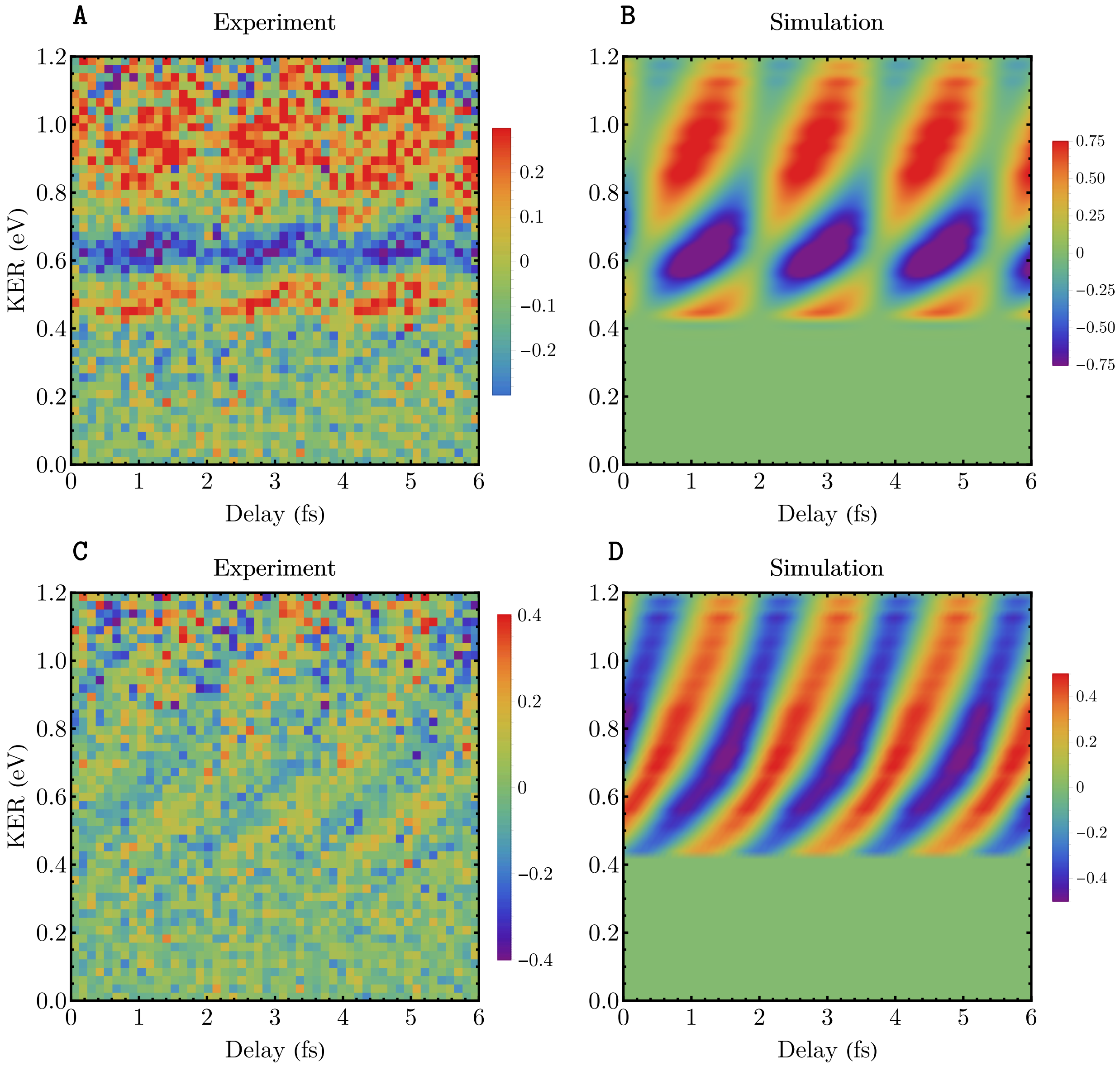

Fig. S 12: **Time-dependent asymmetry parameter A for odd bands**: A and C Experiment, B and D simulation based on the model. The model includes the discussed main three quantum paths. The values of alpha and beta are obtained using the fit to the experimental data. A and B: asymmetry parameter as a function of the time-delay between the XUV and IR pulses. C and D: asymmetry parameter minus the mean time-independent part ($A - \langle A \rangle$). Experiment and simulation are in fairly close agreement.

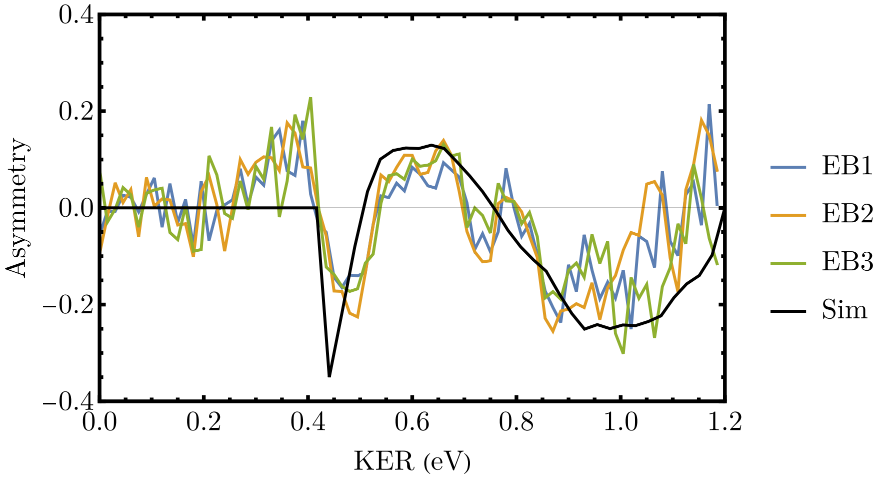

Fig. S 13: Experimental time-integrated even-band asymmetry parameter as a function of KER. The black curve shows the asymmetry A based on Eq. S.28 with $|g_1 + g_2| = \alpha_e$ and $|b1| = \beta_e$.

and the asymmetry parameter becomes:

$$A_{even}(\tau) = \frac{-2(|g_2||b_1|\cos(\Delta\phi_{g2,b1}) + |g_1||b_1|\cos(\Delta\phi_{g1,b1}))}{|g_1|^2 + |g_2|^2 + |b_1|^2 + 2|g_1||g_2|\cos(\Delta\phi_{b1,b2})}, \tag{S.28}$$

with complex amplitudes:

$$g_1 = FC_{gs}\mathrm{e}^{\mathrm{i}\theta_{gs}} \times M_{gs}^{(2)}, \tag{S.29}$$

$$g_2 = FC_{gs}\mathrm{e}^{\mathrm{i}\theta_{gs}} \times M_{gs}^{(2)}, \tag{S.30}$$

$$b_1 = FC_{bs}\mathrm{e}^{\mathrm{i}\theta_{bs}} \times \mathrm{e}^{\mathrm{i}\phi(\tau)} \times M_{bs}^{(1)}. \tag{S.31}$$

The phase difference between the nuclear parts is the same as for the odd bands, $\Delta\Theta_{even} = \Delta\Theta_{odd}$. However, the photoelectron absorbs a different number of IR photons. The phases of the complex amplitudes are:

$$\arg[g1] = \Theta_{gs} - \frac{\pi\ell}{2} + \phi_{q-2}^{XUV} + \phi(\tau) = \Phi_{g1} + \phi(\tau), \tag{S.32}$$

$$\arg[g2] = \Theta_{gs} - \frac{\pi\ell}{2} + \phi_{q}^{XUV} - \phi(\tau) = \Phi_{g2} - \phi(\tau), \tag{S.33}$$

$$\arg[b1] = \Theta_{bs} - \frac{\pi}{2} - \frac{\pi\ell}{2} + \phi_{q-2}^{XUV} + \phi(\tau) = \Phi_{b2} + \phi(\tau). \tag{S.34}$$

With this, the time dependent asymmetry reads:

$$A_{even}(\tau) = \frac{2|g_2||b_1|\sin\big(\Delta\Theta + \Delta\phi^{XUV}_{q,q-2} - 2\phi(\tau)\big) + 2|g_1||b_1|\sin(\Delta\Theta)}{|g_1|^2 + |g_2|^2 + |b_1|^2 + 2|g_1||g_2|\cos\big(\Delta\phi^{XUV}_{q-2,q} + 2\phi(\tau)\big)}, \tag{S.35}$$

and the time-averaged asymmetry parameter is not zero: $\langle A\rangle = \int d\tau A(\tau) \neq 0$.

Fig. 13 shows the experimental time-averaged asymmetry parameter $\langle A\rangle_\tau$ as a function of KER for three even bands (EBs). They correspond to sidebands 18th (EB1), 20th (EB2), and 22st (EB3). The black line is the theoretical curve (Eq. S.35) based on the model explained above.

The final plots for the asymmetry parameter A for even bands are shown in Fig. S14-A. Fig. 14-B shows the corresponding simulated case based on the WKB approximation, the model above and the retrieved α and β parameters. Fig. S14-C and -D shows the asymmetry parameter from -A and -B minus the mean time-averaged contribution $A(\tau) - \langle A(\tau)\rangle$ for both the experiment and theory. Experiment and simulation are in fairly close agreement.

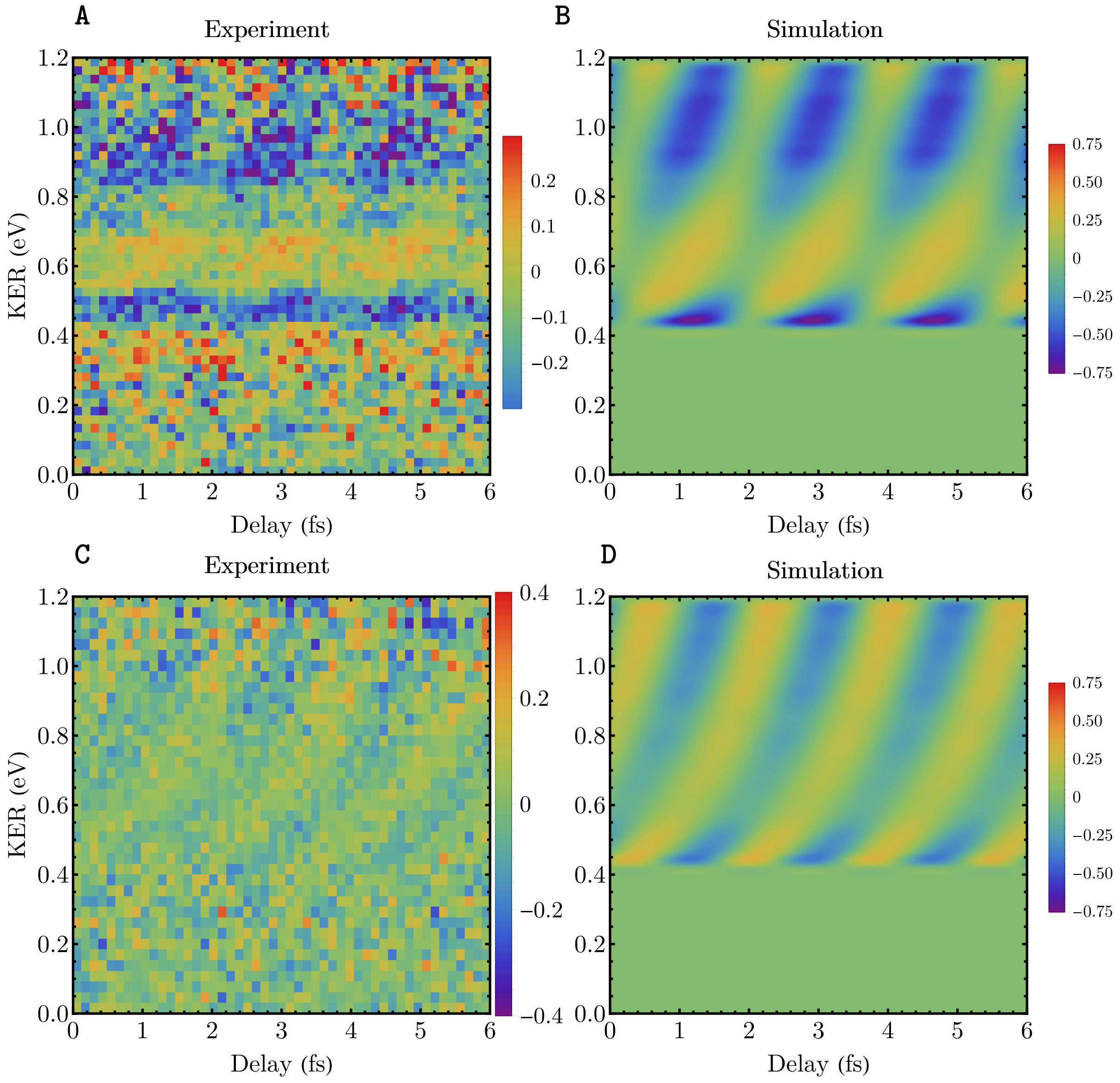

Fig. S 14: **Time-dependent asymmetry parameter A for even bands**: A and C Experiment, B and D simulation based on the model. The model includes the discussed main three quantum paths. The values of alpha and beta are obtained using the fit to the experimental data. A and B: asymmetry parameter as a function of the time-delay between the XUV and IR pulses. C and D: asymmetry parameter minus the mean time-independent part $(A - \langle A \rangle)$. Experiment and simulation are in fairly close agreement.

References

1. J Ullrich, R Moshammer, A Dorn, R Dörner, L Ph H Schmidt, and H Schmidt-Böcking. Recoil-ion and electron momentum spectroscopy: reaction-microscopes. *Reports on Progress in Physics*, 66(9):1463, 2003. 2

2. Farshad Shobeiry. *Attosecond Electron-Nuclear Dynamics in Photodissociation of H2 and D2*. PhD thesis, 2021. 3, 4, 5, 20

3. Richard N. Zare. Photoejection dynamics,. *Mol. Photochem*, 1972. 4

4. Y. M. Chung, E.-M. Lee, T. Masuoka, and James A. R. Samson. Dissociative photoionization of h2 from 18 to 124 ev. *The Journal of Chemical Physics*, 99(2):885–889, 1993. 6

5. Andreas Fischer, Alexander Sperl, Philipp Cörlin, Michael Schönwald, Sebastian Meuren, Joachim Ullrich, Thomas Pfeifer, Robert Moshammer, and Arne Senftleben. Measurement of the autoionization lifetime of the energetically lowest doubly excited state in h2using electron ejection asymmetry. *Journal of Physics B: Atomic, Molecular and Optical Physics*, 47(2):021001, dec 2013. 10

6. Alicia Palacios, Johannes Feist, Alberto González-Castrillo, José Luis Sanz-Vicario, and Fernando Martín. Autoionization of molecular hydrogen: Where do the fano lineshapes go? *ChemPhysChem*, 14(7):1456–1463, 2013. 14

7. Eric E. Aubanel, Jean-Marc Gauthier, and André D. Bandrauk. Molecular stabilization and angular distribution in photodissociation of h2+ in intense laser fields. *Phys. Rev. A*, 48:2145–2152, Sep 1993. 16

8. Manohar Awasthi, Yulian V. Vanne, Alejandro Saenz, Alberto Castro, and Piero Decleva. Single-active-electron approximation for describing molecules in ultrashort laser pulses and its application to molecular hydrogen. *Phys. Rev. A*, 77:063403, Jun 2008. 16

9. Divya Bharti, David Atri-Schuller, Gavin Menning, Kathryn R. Hamilton, Robert Moshammer, Thomas Pfeifer, Nicolas Douguet, Klaus Bartschat, and Anne Harth. Decomposition of the transition phase in multi-sideband schemes for reconstruction of attosecond beating by interference of two-photon transitions. *Phys. Rev. A*, 103:022834, Feb 2021. 16

10. P. M. Paul, E. S. Toma, P. Breger, G. Mullot, F. Augé, Ph. Balcou, H. G. Muller, and P. Agostini. Observation of a train of attosecond pulses from high harmonic generation. *Science*, 292(5522):1689–1692, 2001. 16, 18

11. H.G. Muller. Reconstruction of attosecond harmonic beating by interference of two-photon transitions. *Applied Physics B*, 74(1):s17–s21, 2002. 18
